# The Pathway to China’s Carbon Neutrality Based on an Endogenous Technology CGE Model

**DOI:** 10.3390/ijerph19106251

**Published:** 2022-05-20

**Authors:** Shuang Liang, Xinyue Lin, Xiaoxue Liu, Haoran Pan

**Affiliations:** 1 School of Economics and Resource Management, Beijing Normal University, Beijing 100875, China; alice_liang@mail.bnu.edu.cn (S.L.); lin_xinyue@mail.bnu.edu.cn (X.L.); hrpan@bnu.edu.cn (H.P.); 2 Center for Innovation and Development Studies, Beijing Normal University, Zhuhai 519087, China; 3 School of Economics, Beijing Technology and Business University, Beijing 100048, China

**Keywords:** carbon neutral, endogenous technological progress, computable general equilibrium analysis, economic development, carbon tax, carbon trading

## Abstract

Global warming resulting from greenhouse gas emissions has been a worldwide issue facing humanity. Simultaneously, governments have the challenging task of striking a judicious balance between increased economic growth and decreased carbon emissions. Based on the energy-environment-economy triple coupling (3E-CGE) model, we endogenously integrate climate-friendly technologies into the model’s analysis framework through logic curves and refine and modify the CGE model’s energy use and carbon emission modules. We conduct a scenario simulation and sensitivity analysis on carbon tax, carbon-trading, and climate-friendly technological progress, respectively. The results reveal that carbon tax and carbon trading contribute to reducing carbon emissions in the short-term but achieving the goals of peak carbon and carbon neutrality will cause the collapse of the economic system. In the long-term, climate-friendly technologies are key to achieving the dual carbon goal; the development of such technologies can also stimulate economic development. The best path for China to achieve its dual carbon goals and economic development in the next 40 years involves effectively combining the carbon tax, carbon trading, and a climate-friendly technological progress. Specifically, China can begin trading carbon in high-emissions industries then impose industry-wide carbon taxes.

## 1. Introduction

Recently, global natural disasters caused by climate change have become more frequent, with serious social and economic effects. Climate change has significantly impacted China’s natural ecosystem and socio-economic system. Fossil energy shortages, dramatic price increases, and ecological damage are bottlenecks to future economic growth. In the 40 years of reform during China’s opening-up period, the nation’s rapid economic growth has been accompanied by heavy resource and environmental costs for development due to an over-reliance on resources and factor inputs. On 22 September 2020, China pledged to the world at the general debate of the 75th UN General Assembly that China would scale up its nationally determined contributions, adopt more vigorous policies and measures, and that China aimed to peak carbon dioxide (CO_2_) emissions before 2030 and achieve carbon neutrality before 2060. To achieve this “30–60” target, the Chinese government should adopt various approaches to reduce carbon emissions, including various policy-related mechanisms.

Many realistic development needs suggest that achieving peak carbon and carbon neutrality involves extensive, profound economic and social systemic change, and that China’s energy- and consumption-related, industrial, and regional structures will experience significant adjustments in the next 40 years. Therefore, China urgently requires a more realistic carbon-neutral model for scenario simulations to indicate the nation’s path toward economic transformation. The relevant carbon-emissions models can be divided into 3 categories: (1) top-down models, such as the computable general equilibrium (CGE), dynamic integrated climate-economy, and regional integrated climate-economy models. These analyze the impact of reducing emissions from a macroeconomic perspective, providing useful information on the effects of climate environmental policy implementation [1,2,3]. (2) Bottom-up models, such as the Program of Energy and Climate Economics (PECE-LIU2020) and the low emissions analysis platform energy model, focus on production-level processes and technologies. These simulate energy consumption and technologies under different target scenarios from the local equilibrium perspective, providing enhanced insights to examine emissions-reduction sectors and initiatives [4]. (3) Combining the former 2 approaches can result in a comprehensive evaluation model. Such models emerged in the 1990s, predominantly as a result of climate change research, and represented multidisciplinary, policy-oriented application approaches developed for integrated evaluations, such as the China greenhouse gas emissions scenario-analysis model, the regional air pollution information and simulation model from the International Institute for Applied System Analysis, the Stockholm Environment Institute’s clay-and-sand model, and the Imperial College of Science, Technology, and Medicine’s all-scale atmospheric model [5,6]. Such models combine macro- and micro-economic sectors and can examine the economic losses that the economy and society must sustain while meeting specific emissions-reduction targets [7].

Given the imperative concern in implementing a pathway toward carbon neutrality in China at a minimum cost, this paper will first reveal the macroeconomic effects of implementing a carbon policy and technological progress in a different way, using an endogenous technology (3E-CGE) model. From a temporal perspective, this research aims to understand how developing climate-friendly technologies will profoundly impact China’s economic structure. Second, this research will comprehensively study a combination of carbon-based policy and climate-friendly technology in the context of China’s unique socio-economic, energy, and political contexts.

This study also provides the following contributions: first, we establish a 3E-CGE model, for the first time, with dynamic characteristics of the economy-energy system-carbon emission linkage. The model can portray the trajectory of carbon emissions, the required policies and investments in climate-friendly technology development, and the systemic impacts on the national economy over the next 40 years. Second, a logistic curve is introduced in the model to describe the cycle of technological improvement. The model with endogenous technological progress is closely associated with the investment in the energy sector, which can more clearly reflect the cause of technological development and its effect on the economy-energy-environment system. Third, we simulate the trade-off effects of decreased carbon emissions policy and increased technological progress, and provide the most appropriate development path for China, that is to use a combination of carbon tax and carbon trading policy instruments, while steadily developing climate-friendly technologies.

The remainder of this paper is organized as follows. Section 2 reviews current literature and outlines prior contributions. Section 3 integrates the carbon and technology progress modules with the energy sector to develop a dynamic CGE model. Section 4 discusses peak carbon emissions and the GDP growth rate, and three scenarios will be established and simulated. Section 5 considers the simulation results to analyze the strategies for carbon implementation. Section 6 concludes by proposing several corresponding policy suggestions for China to better realize the “30–60” target and low-carbon transformation.

## 2. Literature Review

The current literature has various paths of discourse regarding carbon neutrality and carbon emission reduction. For example, researchers have focused on the development of energy technologies to reduce carbon emissions [8,9,10,11], as well as carbon taxes and carbon trading [12,13]. A few scholars have also begun to study the long-term dynamics of carbon prices [14]. Further, some CGE models have attempted to simulate development toward carbon neutrality, but most of the existing CGE models consider technology as an exogenous factor and do not adequately describe the role of climate-friendly technologies in the context of carbon neutrality goals. Therefore, this paper considers these previous studies’ results in an attempt to solve this problem.

First, existing literature primarily adopts a “bottom-up” model that focuses on describing the development path of energy technologies. Relatively few studies have addressed carbon neutrality’s impact on China’s macro-economy and micro-economy at the industry level. The Institute of Climate Change and Sustainable Development at Tsinghua University [15] proposed that China should further increase its overall efforts to reduce emissions by promoting breakthroughs in zero- or negative-emissions technologies, strengthening carbon sink-absorption and carbon-removal technologies, and achieving net-zero emissions of all greenhouse gases as soon as possible. Adair Turner et al. [16] comprehensively assessed China’s zero-carbon society to argue that the realization of China’s carbon neutrality vision requires the complete decarbonization of the power-generation sector; maximum electrification of all economic sectors; and the large-scale application of hydrogen and biomass energies as well as carbon capture, utilization, and storage technologies.

Their study details the development path of an energy system to achieve carbon neutrality in China, but only focuses on such economic growth indicators as GDP, consumption, investments, and import and export trades. Academic discussions on carbon emissions and economic growth have long focused on testing whether the environmental Kuznets curve hypothesis is tenable [17,18,19,20,21]. However, this curve cannot accurately predict the future relationship between the economy and environment, and any study of carbon neutrality in China should not be limited to an economic growth perspective. How can we better optimize China’s economic structure? Existing literature has yet to comprehensively analyze industrial restructuring, development modes among non-energy industries, and changes in consumption patterns under carbon neutrality goals; further, any attempts in prior literature are inconsistent. For example, Liu and Cai [22] studied the impacts of technological progress, changes to the industrial structure, and price changes on the intermediate consumption level in the national economy through direct consumption-coefficient and intermediate demand consumption matrices as applied to three major industrial sectors.

Second, the path toward carbon neutrality in China is still unclear, and academic community is still exploring the most feasible solution. According to the Coase theorem, the government can correct negative externalities by internalizing environmental costs into the production and consumption costs of relevant emitters through policy measures or market actions. At present, generally accepted carbon pricing methods primarily include carbon taxes and trading; however, most related studies on these topics are short-term [23,24,25,26,27,28,29,30,31,32]. For example, Galeotti and Larsen [17] found that carbon taxes have not performed well in Norway, as a lower energy intensity and changes in the nation’s energy structure led to a 14% decrease in carbon dioxide emissions, while a carbon tax reduced carbon dioxide emissions by only 2%. Mardones and García [13] found that implementing a carbon tax (US$5 to US$131 per ton) led to no significant decrease in agricultural emissions. Yang et al. [33] used the CGE model to study different carbon tax prices’ impacts on China’s provincial economy. The results reveal that levying a Chinese yuan (CNY) 40 carbon tax effectively reduced CO_2_ emissions and negative effects.

The latest related research using the CGE model still focuses on examining economic impacts and decreases in carbon emissions through implementing appropriate carbon tax rates [34]. Nong et al. [35] analyzed the impacts of one carbon emissions-trading system on the environment and economy to demonstrate that such a system was effective in Vietnam, as it successfully decreased emissions at a low cost. Choi et al. [36] used the CGE model to analyze the usefulness of South Korea’s carbon-trading policies and observed that the best carbon price to promote emissions trading is US$9.14 per ton. Some existing studies on the long-term dynamics of carbon prices have not been conducted in a carbon neutrality context. Ntombela et al. [14] used a dynamic CGE model to assess carbon tax policies’ potential impacts on South Africa’s agriculture and food sectors, among others. The results indicate that implementing a carbon tax by 2035 will reduce carbon dioxide emissions by 33% compared to a baseline scenario but would result in welfare losses of US$5.9 billion. Zhou et al. [37] used the economic-energy-environment CGE model to analyze the impacts of different carbon tax rates on China’s economy and agriculture from 2020 to 2050. They noted that a carbon tax’s short-term effects in reducing carbon dioxide emissions are superior to the long-term effects of reducing carbon intensity and improving energy efficiency.

However, some scholars have illustrated that the carbon tax has no significant impacts, but it also has dual emissions-reduction effects. Shi et al. [38] evaluated carbon trading’s impact on China through the CGE model to reveal that a carbon-trading mechanism can effectively reduce both carbon and energy intensity and promote processes to conserve energy and reduce emissions in China. This mechanism also has certain simultaneous, negative impacts on economic output. Vera and Sauma [39] pointed out that while a carbon tax as a tax system can promote energy conservation and decreased emissions, it will inevitably redistribute wealth to a certain extent, affecting macroeconomic costs and social welfare. Lin and Jia [34] analyzed the carbon tax system’s effects on energy, the environment, and the economy to discover that the carbon tax rate follows the “law of increasing marginal emissions reduction.” Considering the driving factors to decrease emissions, the general consensus at this stage is that technological progress, improved efficiency, and adjustments to the energy structure are the main driving factors to decrease carbon dioxide emissions [40].

Third, most studies of existing CGE models regard climate-friendly technologies as a given exogenous factor [41,42], which does not fully illustrate the role of climate-friendly technology given the goal of carbon neutrality, thus failing to establish an effective link between the economy, energy, and the environmental system. Climate change research has mostly incorporated the theory of exogenous technological progress, with improved energy efficiency used to represent technological progress. The flaw in this theory is that technological progress is regarded as a black-box operational process unaffected by price-induced and innovative activities, which cannot truly reflect the process of technological progress. Another application of exogenous technological progress theory in the energy technology field is a purported “backstop” technology. This typically refers to a technology that has been developed but has not yet entered the market or will enter the market at a certain period in the future, thus changing the energy technology progress. Similarly to automatic energy efficiency index (AEEI), Löschel [43] argues that this assumption is unsatisfactory, because it cannot accurately predict a technology’s future details and costs in the future.

The recent endogenous economic growth theory posits that long-term economic growth comes from the positive external effects of knowledge accumulation, and the economic system can influence this accumulation of knowledge by adjusting R&D investments, thereby promoting technological progress within the system [44,45,46]. Generally, models based on exogenous technological progress theory assume that technological progress is a definite time trend, while models based on endogenous technological progress theory regard knowledge accumulation as a form of capital accumulation that represents technological progress [47]. The application of endogenous technological progress theory in climate change takes many forms. Existing literature using endogenous technologies to study low-carbon and economic growth includes dynamic input-output models and CGE models [48]. Pan [49,50] regards the input-output coefficient as the result of combining a specific technology in an early stage and an existing specific technology and believes that technological progress is the process of alternately updating old and new technologies. The research designed a dynamic input-output model and introduced technological progress and diffusion as endogenous variables; R&D investments were used to drive new technological progress along a logical curve until maturity and decay. Further, fixed assets installation investments were used to diffuse new technologies and eliminate old technologies. The result of this alternate adjustment of new and old technologies within the industry promoted an industrial transformation, which then led to changes in the entire industrial structure.

This method was subsequently applied to study the replacement of fossil and non-fossil energy technologies in China’s power industry, as low-carbon energy technologies will significantly change China’s power industry structure and economic structure in the future. Wang et al. [51] constructed an endogenous technological progress CGE model to study China’s climate change issues. The model focuses on technological progress in energy and environment modules. According to the theory of endogenous technological progress, the model separates the R&D matrix from the intermediate input sector, adds a knowledge capital input to the factor sector, and increases R&D investments in the final demand column. Kristkova et al. [52] introduced public R&D investment as a sector in the CGE model to study its effect on agricultural productivity and food security.

Current works on endogenous technological progress involving climate change and economic growth primarily considers R&D investments—or specifically, separates the R&D department—or conducts research with R&D investments as one element. This method not only creates spillover effects and inconsistency between social and private benefits, but also controls the development path of technological progress; thus, it is more suitable for short-term research [53]. Judging from a 40-year simulation of China’s developing carbon neutrality, technological progress has significantly impacted both carbon neutrality and economic growth. Therefore, a more suitable long-term endogenous technological progress model is needed to explore China’s decreased carbon emissions and path of development.

Generally, research using the CGE model to simulate carbon tax and trading policies and evaluate their role in decreasing carbon emissions has been relatively mature, and several discussions have included the Kuznets curve of carbon emissions and economic growth. However, these studies lack systematic, long-term characteristics, and rarely involve the mechanisms of economic structural adjustments and their specific effects on carbon dioxide emissions. Most of these also ignore endogenous technological progress’ impacts on the economy-environment system. Additionally, relevant research on China’s carbon neutrality is still in an early stage, and primarily focuses on practical approaches, technologies, and standards. Detailed empirical research is required, and especially studies of carbon neutrality targets’ impact on economic transformations using a macroeconomic model. Therefore, this study is based on realistic economic theories, methods, and data from China, which has proposed an integrated energy system comprised of regional economic and social data. This system can be used to develop an integrated energy-environment-economy CGE (3E-CGE) model for China that evolves an endogenous, climate-friendly technological innovation process to study climate change policies. This study will focus on the trajectory of carbon emissions and the policies and investments required for climate-friendly technological developments. It will also examine the systemic impacts on the national economy, including changes to the industrial structure and energy system, and adjustments to consumption patterns from 2020 to 2060. Ultimately, this work will explore the optimal development path for China under “peak carbon” and “carbon neutrality” constraints.

## 3. Model and Methods

### 3.1. The Model’s Basic Structure

**Figure 1:**
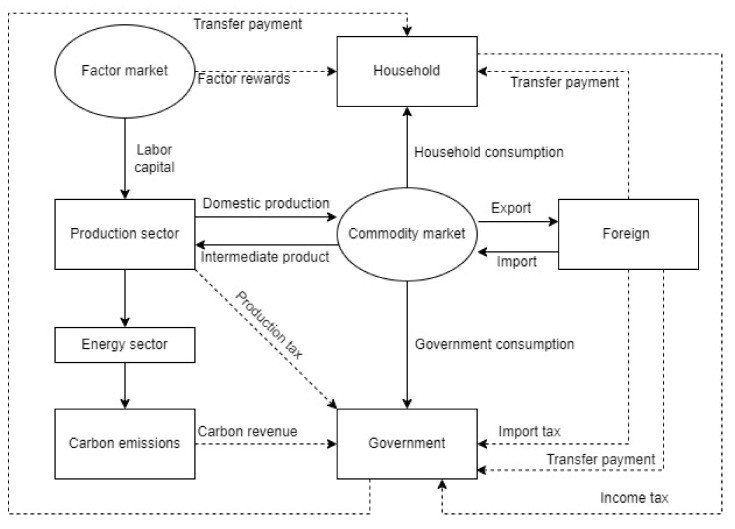
The CGE model framework diagram.

We construct a computable general equilibrium model of China’s energy and carbon dynamics that includes the following modules: production, energy, revenue and expenditures, trade, carbon, dynamic, climate-friendly technology, and closure (Figure 1). The model describes a closed-loop system, with each module interlocking and interacting through price and output variables. In the model, “PS” and “CC” denote the industry and product dimensions, respectively. Here, we only briefly describe the model structure.

#### 3.1.1. Production Module

**Figure 2:**
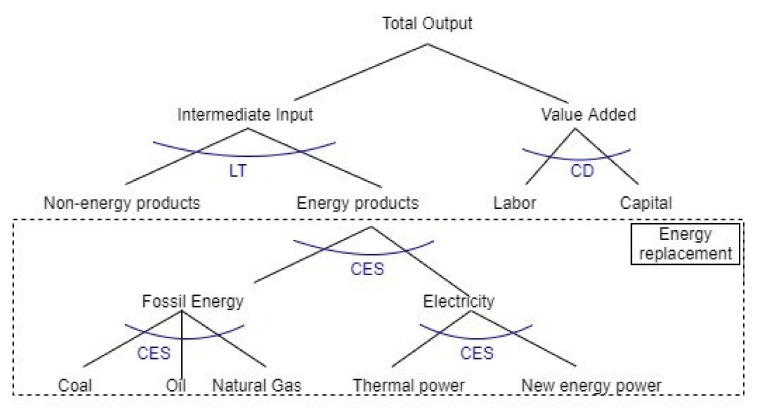
Production structure diagram.

The production module describes the relationship between the product input and output in the Chinese production sector. This model assumes that the market is completely competitive, sectoral output is determined by market equilibrium conditions, and production decisions are made in accordance with the principle of cost minimization. To reflect and address the more complex substitutive relationship between multiple inputs, the production module uses a multi-layer, nested design form (Figure 2). The first layer of nesting is solved by the intermediate input and composite elements through the CES function. The production function of the combination of intermediate input and added value is as follows:

(1) $$X\left(\mathrm{PS}\right)=\mathrm{AP}\left(\mathrm{PS}\right)\cdot {\left[\beta \left(\mathrm{PS}\right)\cdot {U(\mathrm{PS})\;}^{\rho \left(\mathrm{PS}\right)}+\beta \prime \left(\mathrm{PS}\right)\cdot {V(\mathrm{PS})\;}^{\rho \left(\mathrm{PS}\right)}\right]}^{\frac{1}{\rho \left(\mathrm{PS}\right)}}$$

where AP (PS) is the scale coefficient of the sector; U (PS) and V (PS) represent the intermediate goods input aggregation and collected bundle of factors from sector PS, respectively; β (PS) and β’ (PS) denote the shared parameter representing intermediate goods and factors from sector PS, respectively; ρ (PS) is the substitution parameter; and X (PS) is the production function from combining intermediate input and added value. The second layer consists of two parts. The first compounds the intermediate input U(PS) through the LT function, and the second compounds the capital-labor-land element bundle V(PS) through the CES function. The formula is as follows:

(2) $$\;U\left(\mathrm{PS}\right)=\sum_{\mathrm{CC}}\mathrm{ut}\left(\mathrm{CC},\mathrm{PS}\right)\cdot \mathrm{XX}\left(\mathrm{CC},\mathrm{PS}\right)$$

(3) $$V(\mathrm{PS})=\mathrm{AV}\left(\mathrm{PS}\right)\cdot {\left[\alpha \left(\mathrm{PS}\right)\cdot {L(\mathrm{PS})\;}^{\rho 2\left(\mathrm{PS}\right)}+\alpha \prime \left(\mathrm{PS}\right)\cdot {K(\mathrm{PS})}^{\rho 2\left(\mathrm{PS}\right)}\right]}^{\frac{1}{\rho 2\left(\mathrm{PS}\right)}}$$

where XX (CC, PS) represents the intermediate production input; ut (CC, PS) denotes the shared parameter of commodity CC used by sector PS in the LT function; AV (PS) is the total factor productivity; α(PS) and α’ (PS) are the input and shared parameters of labor factor L (PS) and capital factor K (PS) as used by sector PS in the CES function, respectively; and ρ2 (PS) is the substitution coefficient.

The third layer describes the energy substitution portion. First, the energy product bundle is formed by combining both energy and power products through the CES function.

(4) $$\mathrm{UEE}(\mathrm{PS})=\mathrm{APEE}\left(\mathrm{PS}\right)\cdot {\left[\gamma \left(\mathrm{PS}\right)\cdot {\mathrm{UEN}(\mathrm{PS})}^{\rho 3\left(\mathrm{PS}\right)}+\gamma \prime \left(\mathrm{PS}\right)\cdot {\mathrm{UEP}(\mathrm{PS})}^{\rho 3\left(\mathrm{PS}\right)}\right]}^{\frac{1}{\rho 3\left(\mathrm{PS}\right)}}$$

In the previous formula, UEE (PS) represents the sector’s energy cluster production input; UEN (PS) and UEP (PS) represent the sector’s fossil energy and power cluster production inputs, respectively; APEE (PS) denotes the scale coefficient; γ (PS) and γ’(PS) are the shared parameters of sector PS using both fossil energy and power products in the CES function; and ρ3(PS) is the substitution coefficient.

The fourth layer is the production structure of fossil energy products and power products. The energy products on the left side of Figure 2 are composed of coal, oil, and natural gas through the CES function, while the power products on the right are composed of thermal and new energy power.

(5) $$\mathrm{UEN}\left(\mathrm{PS}\right)=\mathrm{APEN}\left(\mathrm{PS}\right)\cdot {\left[\underset{\mathrm{EN}}{\sum }\varphi \left(\mathrm{EN},\mathrm{PS}\right)\cdot \mathrm{QXEN}{\left(\mathrm{EN},\mathrm{PS}\right)}^{\rho 4\left(\mathrm{PS}\right)}\right]}^{\frac{1}{\rho 4\left(\mathrm{PS}\right)}}$$

(6) $$\mathrm{UEP}\left(\mathrm{PS}\right)=\mathrm{APEP}\left(\mathrm{PS}\right)\cdot {\left[\underset{\mathrm{EP}}{\sum }\theta \left(\mathrm{EP},\mathrm{PS}\right)\cdot \mathrm{QXEP}{\left(\mathrm{EP},\mathrm{PS}\right)}^{\rho 5\left(\mathrm{PS}\right)}\right]}^{\frac{1}{\rho 5\left(\mathrm{PS}\right)}}$$

where QXEN(EN,PS) and QXEP(EP,PS) represent the sector’s investment in fossil energy and power products, respectively; APEN(PS) and APEP(PS) are the scale coefficients; φ(EN,PS) denotes the shared parameter of coal, oil, and natural gas; θ(EP,PS) represents the shared parameter of thermal and new energy power product inputs; and ρ_4_(PS) and ρ_5_(PS) are substitution coefficients.

#### 3.1.2. Revenue and Expenditure Module

The revenue and expenditure module includes two main bodies—residents and the government—which use the Cobb-Douglas utility function to maximize utility under the constraints of the income function. Residents’ income comes from labor income, capital remuneration, and government transfer payments, and is used for consumption or savings after paying income taxes. Government revenue comes from production, consumption, value-added, import, and income taxes; the government’s expenditures include the purchase of goods, transfer payments, and government savings.

#### 3.1.3. Trade Module

The goods in the commodity supply come from domestic production and imports, which are used for final demand and intermediate consumption. To achieve the lowest consumption cost, rational consumers will optimize a combination of domestic and imported goods in their purchases; between the two, the Armington condition [54] is met. Specifically, an incomplete substitution occurs between imported and domestic products. In terms of price selection, this model assumes that the imported goods’ price is exogenously provided, the imported goods’ price is determined by the international market price, and the importer is the price taker:

(7) $$\mathrm{QC}\left(\mathrm{CC}\right)=\mathrm{AA}\left(\mathrm{CC}\right)\cdot {\left[\delta \left(\mathrm{CC}\right)\cdot \mathrm{QD}{\left(\mathrm{CC}\right)}^{\mathrm{sa}\left(\mathrm{CC}\right)}+\;\delta \prime \left(\mathrm{CC}\right)\cdot \mathrm{IMP}{\left(\mathrm{CC}\right)}^{\mathrm{sa}\left(\mathrm{CC}\right)}\right]}^{\frac{1}{\mathrm{sa}\left(\mathrm{CC}\right)}}$$

where AA(CC) is the scale coefficient; δ(CC) and δ’(CC) represent the shared parameters; sa(CC) represents the conversion elasticity; QD(CC) and IMP(CC) represent domestically produced self-sale and imported goods, respectively; and QC(CC) represents domestic goods. Similarly, assuming that all countries in an international market sector are a smaller size, export commodities are also determined exogenously by international market price. The total domestic output is sold at home and abroad in accordance with the principle of profit maximization. Manufacturers will optimize the combination between domestic sales and exports, and this combined relationship is allocated through the fixed conversion-elastic (CET) function.

(8) $$Q\left(\mathrm{CC}\right)=\mathrm{AT}\left(\mathrm{CC}\right)\cdot {\left[\epsilon \left(\mathrm{CC}\right)\cdot \mathrm{QD}{\left(\mathrm{CC}\right)}^{\mathrm{st}\left(\mathrm{CC}\right)}+\epsilon^{\prime }\left(\mathrm{CC}\right)\cdot \mathrm{EXP}{\left(\mathrm{CC}\right)}^{\mathrm{st}\left(\mathrm{CC}\right)}\right]}^{\frac{1}{\mathrm{st}\left(\mathrm{CC}\right)}}$$

In Formula (8), AT(CC) is the scale coefficient; ϵ(CC) and ϵ’(CC) denote the shared parameters; st(CC) is the conversion elasticity; QD(CC) and EXP(CC) are the domestically produced self-sale and exported goods, respectively; and Q(CC) represents domestically produced goods.

#### 3.1.4. Dynamic Module

The variables in dynamic equations can be roughly divided into two categories. The first category is exogenous growth, which is represented by changes in the labor supply. This model uses a recursive dynamic mechanism, that is, through the dynamic changes in labor force growth $\mathrm{LST}\left(\mathrm{TH}\right)$ and capital accumulation KST(TH) to modify the model. In the long-term, the labor force and population maintain the same proportion of growth and decline, and the population is hardly affected by economic policies. Therefore, such variables in the dynamic CGE model are generally exogenous, with the core equation is as follows:

(9) $$\mathrm{LST}\left(\mathrm{TH}+1\right)=\left[1+\mathrm{gpop}\left(\mathrm{TH}\right)\right]\cdot \mathrm{LST}\left(\mathrm{TH}\right)$$

where LST(TH + 1) and LST(TH) represent the labor supply in period *t* + 1 and period *t*, respectively; and gpop(TH) represents the population growth rate in period *t*. The data comes from *World Population Prospects 2017* released by the Population of the UN Department of Economic and Social Affairs. The other category is capital control variables. The capital stock growth rate is driven by investments, and the size of these investments is affected by the rate of return. Among them, each department’s labor force in the base year is given exogenously. The current capital stock is equal to the previous capital stock, plus new capital, and minus depreciation. The distribution of new capital among different sectors uses the CET function to maximize capital gains:

(10) KST(TH+1)=[1+gk(TH)]·[(1−dep)·KST(TH)+INVPS(TH)]

where KST(TH) and KST(TH + 1) represent the capital stock in period *t* and period *t* + 1, respectively; INVPS(TH) denotes the total investment in period *t*; dep is the depreciation rate of macroeconomic capital; and gk(TH) represents the growth rate of capital in period *t*. This model is calculated based on the GDP forecast data from the Organization for Economic Co-operation and Development (OECD).

### 3.2. Carbon Module

#### 3.2.1. Carbon Tax Module

In this model, the carbon tax—which involves collecting carbon emissions in the production-end sector and energy products in the consumption end—is exogenous. Carbon tax revenue is calculated by multiplying carbon tax by carbon emissions, namely:

TXCO2(TH) = TRCO2(TH) ∗ [TCO2(TH) + FDCO2(TH)](11)

where TRCO2(TH) represents the carbon tax price; TXCO2(TH) is the total carbon tax revenue; and TCO2(TH) and FDCO2(TH) represent the total carbon dioxide emissions at the production and consumption end, respectively. As a new cost, carbon tax is included in the pricing formula for energy products at the production and consumption end, respectively:

(12) PQXEN(TH)=PQXEN0(TH)+TRCO2EN(TH)

(13) PCQXN(TH)=PCXEN0(TH)+TRCO2EN(TH)

where TRCO2EN(TH) represents the carbon tax price per unit of energy, PQXEN(TH) and PCQXN(TH) represent the prices of energy products with carbon prices at the production and consumption end of period *t* when carbon tax is imposed, respectively. Further, PQXEN0(TH) and PCXEN0(TH) represent the production and consumption prices of energy products without carbon costs, respectively.

#### 3.2.2. Carbon Trading Module

The carbon emissions trading plan is the same as the market plan for other commodities, as carbon emission credits are regarded as commodities. However, the government controls the number of carbon emissions rights; in the carbon-trading market, the total supply of carbon emission rights will be determined according to the government’s emissions reduction targets. The setting of the carbon cap primarily depends on the corresponding emissions reduction target, with the specific formula set as follows:

(14) $$\mathrm{Carbon}\left(\mathrm{TH}\right)=\underset{\mathrm{PS}}{\sum }\left[1-\mathrm{tcer}\left(\mathrm{PS},\mathrm{TH}\right)\right]\cdot \mathrm{CO}\;2\mathrm{ref}\left(\mathrm{PS},\mathrm{TH}\right)$$

where $\mathrm{Carbon}\left(\mathrm{TH}\right)$ represents the carbon allowance, or the total carbon emissions supply in the carbon-trading market at time *t*; $\mathrm{CO}\;2\mathrm{ref}\left(\mathrm{PS},\mathrm{TH}\right)$ represents various industries’ benchmark carbon emissions during period *t*; and $\mathrm{tcer}\left(\mathrm{PS},\mathrm{TH}\right)$ is the sectoral carbon emission reduction rate set by the government according to its emissions reduction target. The formula for total carbon emissions is as follows:

(15) $$\mathrm{TCO}\;2\left(\mathrm{TH}\right)=\underset{\mathrm{PS}}{\sum }\mathrm{CO}\;2\left(\mathrm{PS},\mathrm{TH}\right)$$

where TCO2(TH) represents the total carbon emissions of all industries in period *t*; and CO2(PS, TH) represents different industries’ carbon emissions, with prices in the carbon-trading market determined by supply and demand. From production cost perspective, an increase in carbon costs in the energy sector will increase energy prices, and using energy as an intermediate input will increase the cost of using carbon-containing energy products. As an alternative, the cost of using low- and non-carbon energy products is relatively lower, with the following carbon cost formula:

(16) $$\mathrm{PQXEN}\left(\mathrm{TH}\right)=\mathrm{PQXEN}0\left(\mathrm{TH}\right)+\mathrm{PCO}\;2\mathrm{EN}\left(\mathrm{TH}\right)$$

where PQXEN(TH) in carbon trading represents the price of energy products with carbon prices in period *t*; PQXEN0(TH) represents the production price without carbon costs; and PCO2EN(TH) represents the carbon price per unit of energy. When allocating carbon trading, the government will also give enterprises a partial emissions exemption, as businesses with such an exemption will not need to pay these costs. The formula for this exemption is calculated as follows:

(17) $$\;\mathrm{TFP}\left(\mathrm{TH}\right)=\underset{\mathrm{PS}}{\sum }\mathrm{fp}\left(\mathrm{PS}\right)\cdot \mathrm{CO}\;2\mathrm{ref}\left(\mathrm{PS},\mathrm{TH}\right)=\underset{\mathrm{PS}}{\sum }\mathrm{FP}\left(\mathrm{PS},\mathrm{TH}\right)$$

(18) $$\mathrm{ET}\left(\mathrm{PS},\mathrm{TH}\right)=\mathrm{TCO}\;2\left(\mathrm{PS},\mathrm{TH}\right)-\mathrm{FP}\left(\mathrm{PS},\mathrm{TH}\right)$$

where TFP(TH) represents the total free allocation quota in period *t*; FP(PS,TH) represents various industries’ free emissions in period *t*, and ET(PS,TH) represents the carbon quota that can be used for inter-sectoral transactions. According to this formula, when the free allocation ratio $\mathrm{fp}\left(\mathrm{PS}\right)$ in the total quota decreases, carbon-intensive industries must purchase more carbon emissions to ensure production and operation. Carbon revenue equals the carbon price per unit of CO2 multiplied by the carbon emissions excluding the exemption, or:

(19) $$\mathrm{TXCO}\;2\left(\mathrm{TH}\right)=\mathrm{PCO}\;2\left(\mathrm{TH}\right)\cdot \left[\mathrm{TCO}\;2\left(\mathrm{TH}\right)-\mathrm{TFP}\left(\mathrm{TH}\right)\right]$$

where $\mathrm{PCO}\;2\left(\mathrm{TH}\right)$ represents the carbon price per unit of CO2.

### 3.3. Technological Progress Module

Climate-friendly technologies include not only technical means for the energy sector to improve energy efficiency, but also technical means for the end consumer and other industrial sectors to reduce carbon dioxide emissions. By introducing the non-carbon energy investment share NClindex (TH), this module creates an endogenous logic curve describing the state of climate-friendly technological progress in the entire system. According to Pan and Kohler [50], the specific formula is as follows:

(20) $$\mathrm{SNCT}\left(\mathrm{TH}\right)=\mathrm{LGCd}+\frac{\mathrm{LGCa}}{{\left\{1+\mathrm{LGCc}\cdot \mathrm{EXP}[-\mathrm{LGCb}\cdot \left(\mathrm{NUMYEAR}\left(\mathrm{TH}\right)-\frac{\mathrm{LGCm}}{\mathrm{NCIindex}\left(\mathrm{TH}\right)}\right)]\right\}}^{\frac{1}{\mathrm{LGCc}}}}$$

where SNCT(TH) denotes the share of climate-friendly technology, which is calculated from the following parameters: LGCa, or saturation; LGCb, or the average growth rate; LGCc, or the acceleration in growth; LGCd, or the initial level; LGCm, or the time to maximum growth; NUMYEAR(TH), or the year; and NClindex(TH), or the non-carbon energy investment ratio. where SWSNCT denotes the change in climate-friendly technology, indicated as either zero or one; when SWSNCT equals one, Formulas (21) is run. Changes in the share of climate-friendly technologies will change the carbon emissions coefficient, which will affect the entire society’s carbon dioxide emissions. As noted in Formulas (21), the changes in VcofCO2CC(CC,PS,TH) is calculated as follows:

(21) $$\mathrm{VcofCO}2\mathrm{CC}\left(\mathrm{CC},\mathrm{PS},\mathrm{TH}\right)=\left\{\begin{array}{ll} \mathrm{cofCO}\;2\mathrm{CC}\left(\mathrm{CC},\mathrm{PS},\mathrm{TH}\right) & \;\mathrm{TH}=1\\ \mathrm{VcofCO}\;2\mathrm{CC}\left(\mathrm{CC},\mathrm{PS},\mathrm{TH}-1\right)\cdot \left[1-\mathrm{SNCT}\left(\mathrm{TH}-1\right)\cdot \mathrm{SWSNCT}\left(\mathrm{TH}-1\right)\right] & \;\mathrm{TH}\;>\;1 \end{array}\right.$$

where cofCO2CC(CC,PS,TH) denotes the carbon emissions coefficient.

### 3.4. Closing Module

To retain the model with a unique solution, the CGE model must set micro- and macro-closures to ensure that the constraint conditions are consistent with the number of endogenous variables. The economy’s market equilibrium solves the equilibrium price, and the equilibrium price is determined by solving the nonlinear equation system. This includes the intermediate and final demand equations as well as the calculation of residential and government income, savings and investments, and the trade balance. This model uses neoclassical closures, or by setting closures in commodity and factor markets, institutional revenues and expenditures, foreign markets, and investment savings. It specifically includes: (1) the commodity and factor market equilibrium; (2) residents’ total consumption, which equals their disposable income minus savings; and total government savings, which equals the government’s income minus consumption and transfer payments to residents; (3) total investments equal total savings; and (4) the difference between imports and exports equals any foreign investments.

### 3.5. Model Data

The input-output analysis method created by Leontief [55] provides the possibility for modern economics to move towards quantitative analyses. In a checkerboard-like table, the relationships between production, the factor input, consumption, investments, and trade are clearly expressed as quantities, and the complete, overall picture and structure of the flow of products in the economic system is revealed. Part of the relationship between industrial sectors constitutes the core of the input-output table, reflecting the mutual influence and interdependence among various industrial sectors. Stone [56] extended the input-output analysis to institutional departments and established income accounts to record the product and income flows as well as transfers of income between institutions. The subsequent social accounting matrix system has become the necessary database to model the current computable general equilibrium model and other large-scale quantitative economic structural models.

As the input-output table for 2017 depicts, in the latest data released by the National Bureau of Statistics at the beginning of this research, 2017 is selected as this study’s base year. The model’s main data includes the following three types: First, the China Social Accounting Matrix (SAM) provides a foundation for the CGE model (Table 1). According to the Input-Output Table of China’s 149 Sectors in 2017, we merged then split the energy input-output tables of 29 sectors, including coal, oil, natural gas, thermal, and new energy power. The fiscal and taxation data in the social accounting matrix comes from the *2018 Tax Yearbook* and *2018 Fiscal Yearbook*. Table 1 also displays the resulting macro-social accounting matrix. Second, the exogenous elasticity of substitution includes that between inputs in the production function, the substitution elasticity between imported and domestic products from the CES function within the foreign trade module, and the substitution elasticity between exports and domestic products in the CET function. Our data is derived from the Global Trade Analysis Project database. Third, we calculate the carbon dioxide emissions coefficient per sector by calculating the sectoral carbon dioxide emission and energy product consumption. The carbon dioxide emissions data for this calculation comes from China’s Carbon Emission Accounts and Datasets (CEADs) database.

## 4. Constraints and Scenario Settings

### 4.1. Discussion on Peak Carbon Emissions

China’s “30–60” commitment—to peak carbon by 2030 and carbon-neutral production by 2060—adds new constraints to China over the next 40 years, with 2 key factors. One is the apex of peak carbon in 2030, and the other is the GDP growth path until 2060.

**Figure 3:**
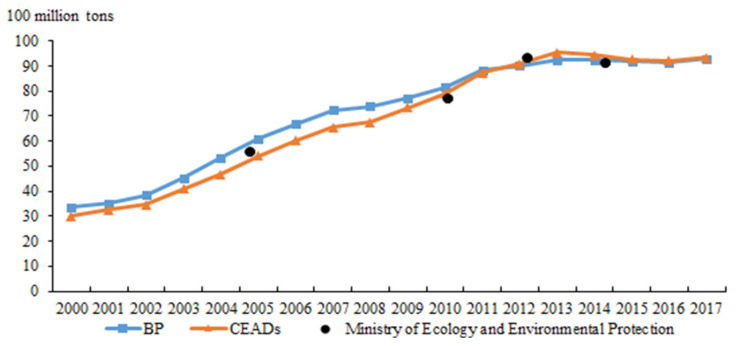
Comparison of China’s carbon emission data in various databases.

Regarding the former, we must first solve the problem of basic carbon emissions data, as the existing carbon emissions database primarily includes data from the CEADs database, British Petroleum (BP), and the Ministry of Ecology and Environment (Figure 3). As the Ministry’s data is not sufficiently continuous, the two time-continuous CEADs and BP databases are more suitable for research. Additionally, the carbon emissions in 2005 (not included in the carbon sink) as calculated using the three databases were 5.4, 6.1, and 5.98 billion tons, respectively. In 2014, the 3 databases’ carbon emissions were 9.44, 9.24, and 10.28 billion tons, respectively. In 2017, the statistical carbon emissions for CEADs and BP were 9.34 and 9.3 billion tons, respectively. The numerical difference between these databases has significantly narrowed in recent years. Considering that the CEADs contains emissions data for 29 industries—which is convenient for a structural analysis and CGE model simulation—we ultimately chose the CEADs for our analysis. To avoid the uncertainty caused by the significant differences between the early carbon emissions data found in different databases, we selected the carbon emissions from the 2017 CEADs (9.34 billion tons) as our calculation benchmark.

Regarding China’s carbon peak target, President Xi Jinping’s critical speech at the General Debate of the 75th United Nations General Assembly on 22 September 2020 indicated that China will enhance its nationally determined contribution. Moreover, the nation would strive to reach peak carbon dioxide emissions by 2030 and carbon neutrality by 2060, or the “30–60 dual carbon target”. Specifically, the carbon emission intensity in 2030 would decrease by more than 65% compared with that in 2005. Combined with the declining rate of carbon emissions intensity in that year compared with 2005, as recently announced by the State Council, the 2017 carbon intensity was 46% lower than that in 2005. It is estimated that carbon emissions in 2030 will reach 11.7 billion tons; after subtracting 910 million tons of carbon sinks, we predict that net emissions will reach 10.8 billion tons in 2030, which is an approximate median of the peak data calculated by Tsinghua University, the World Resources Institute, and other institutions.

### 4.2. Discussion of the GDP Growth Rate

Our research also considers the growth trends of various production factors, such as capital, labor, human capital, and total factor productivity. We refer to relevant research on China’s economic growth forecast, both domestically and internationally, and the outline of the 14th 5-Year Plan, to assume that GDP will double from 2020 to 2035, and the GDP growth rate will decline from 6% in 2019 at a uniform rate. It can be concluded that the compound GDP growth rate from 2020 to 2030 is 5%. By 2035, China’s total GDP will reach 240 trillion yuan, realizing the nation’s long-term goal of doubling its total economic output in 2020. The compound growth rate of the nation’s GDP from 2040 to 2060 is estimated at 3%.

In summary, China only has 10 years to achieve its peak carbon goal and 30 years to achieve carbon neutrality, which is a much shorter duration than European countries and the United States. Moreover, China’s carbon emissions only have 8% room for improvement, with an average annual growth of 0.77%, and it is highly difficult to maintain a relatively high level of GDP growth. How can China meet this challenging goal? We construct various policy scenarios for a simulation in an attempt to discover the optimal path toward implementation.

### 4.3. Scenario Setting

To quantitatively analyze the different effects of the two previously mentioned paths in the two dimensions of carbon neutrality and economic development, we focus on the scenarios listed in Table 2.

## 5. Results and Analysis

### 5.1. Baseline Scenario Results

**Figure 4:**
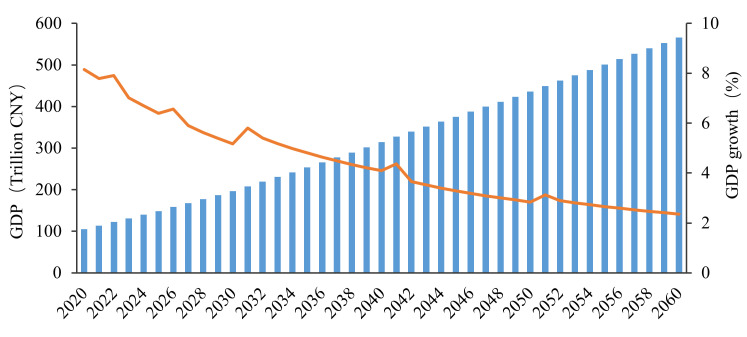
GDP and its annual growth rate in the baseline scenario (BAU) from 2020 to 2060.

**Figure 5:**
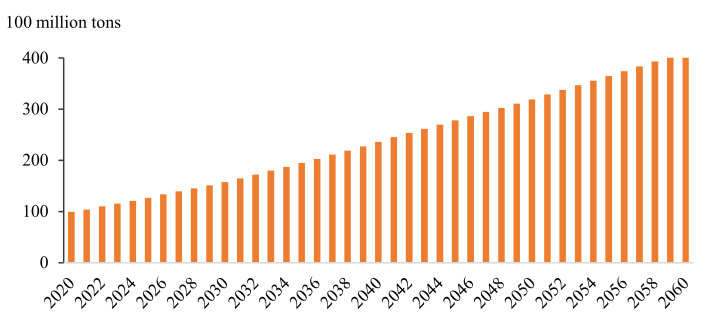
Carbon emissions in the baseline scenario (BAU) from 2020 to 2060.

In the scenario that only considers the goal of doubling the total economic output or per capita income by 2035, the development trend of China’s economy and carbon emissions is that the latter will continue to rise; carbon emissions are expected to reach 41.1 billion tons in 2060 (Figure 4 and Figure 5).

### 5.2. Results of the Carbon Price Policy Scenario

**Figure 6:**
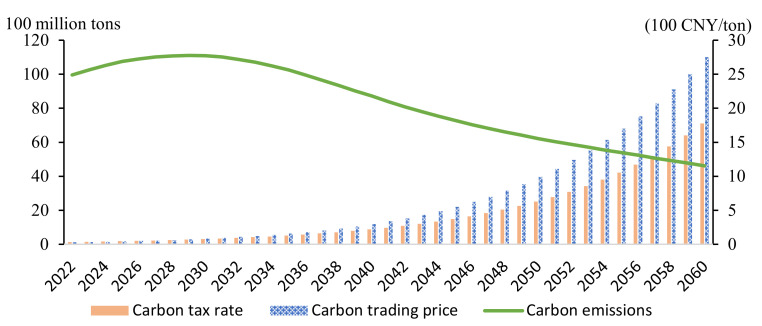
Carbon price and carbon emissions.

Most people intuitively feel that continuously increasing the level of carbon tax can fulfill China’s “30–60” goal. However, this dual carbon goal requires an incredibly high carbon price. As Figure 6 indicates, the carbon tax rate and carbon trading price would increase annually to 2000 CNY per ton, which would collapse China’s economy.

**Figure 7:**
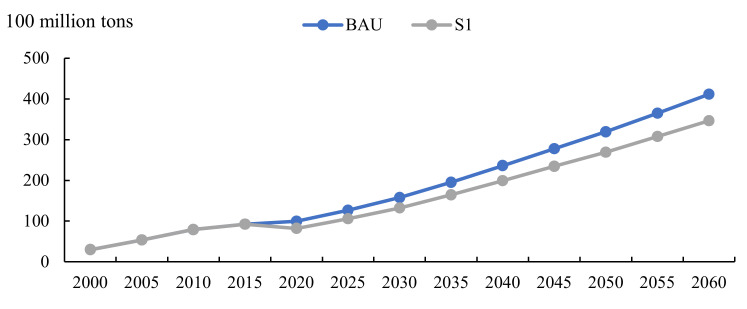
Carbon emissions under carbon price policy S1 from 2020 to 2060.

In this instance, carbon emissions continue to increase, with no inflection point to achieve any peak and neutralization; this can only shift the carbon emissions curve under the BAU scenario downward (Figure 7).

In summary, it can be observed that the carbon-pricing policy can only shift the carbon emissions curve downward, which can reduce emissions in the short-term, but will cause certain economic losses. The higher the carbon price, the greater the economic loss, consistent with the conclusions of most carbon tax and trading policy studies [23,28,34]. While the carbon price policy positively impacts a reduction in emissions, the fundamental transformation of carbon neutralization, this cannot be achieved solely through a carbon tax and carbon trading.

### 5.3. Results of Technological Progress Scenario

As previously mentioned, if we only rely on carbon pricing and do not abandon GDP constraints, it will be difficult to achieve carbon neutrality solely through carbon pricing. Therefore, it is necessary to consider technological progress. Developing climate-friendly technologies will transform the production costs of zero-emissions technology and the structure of energy consumption.

**Figure 8:**
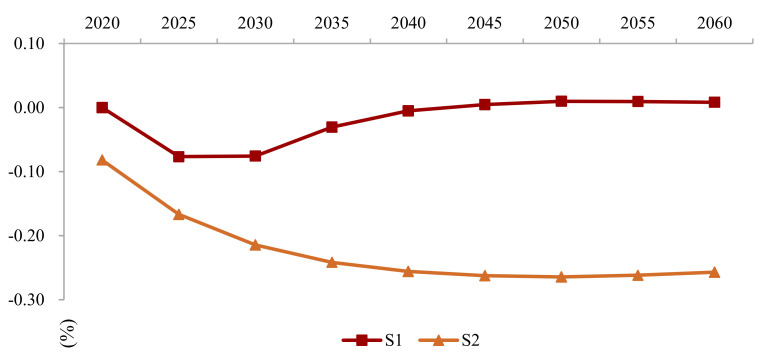
The GDP loss under S1 and S2 scenario compared with the BAU scenario.

Based on the S1 scenario, we levy an CNY 100 carbon tax, implement carbon trading in 8 major industries, and introduce a technology curve. The simulation reveals that the combination of carbon tax, carbon trading, and technological progress should fulfill the dual goals of maintaining growth and carbon neutrality. As Figure 8 indicates, compared with the baseline case, the GDP loss under S1 will be substantial, with a GDP loss of CNY 42 trillion in 2030 and CNY 145 trillion in 2060. In the S2 scenario, the loss of GDP is negligible; even after 2040, the GDP exceeds the baseline, indicating that technological progress has offset or even exceeded carbon pricing’s negative impact on economic growth. Carbon emissions peak in 2030 at 10.9 billion tons, then decreases annually to converge to the net-zero goal.

The simulation results demonstrate that because the power of technological progress has only had a partial influence in the initial stage, carbon taxes and carbon trading still pressure the economy to increase costs. However, this pressure among various industries is alleviated, compared with the situation without technological progress.

**Figure 9:**
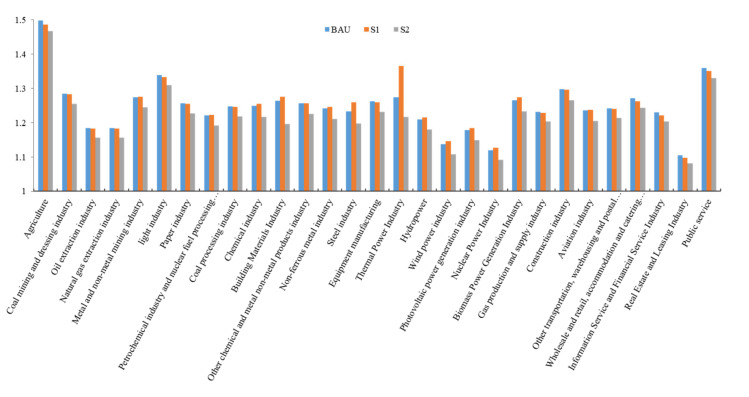
PPI differences in various industries under BAU, S1, and S2 scenarios in 2030.

**Figure 10:**
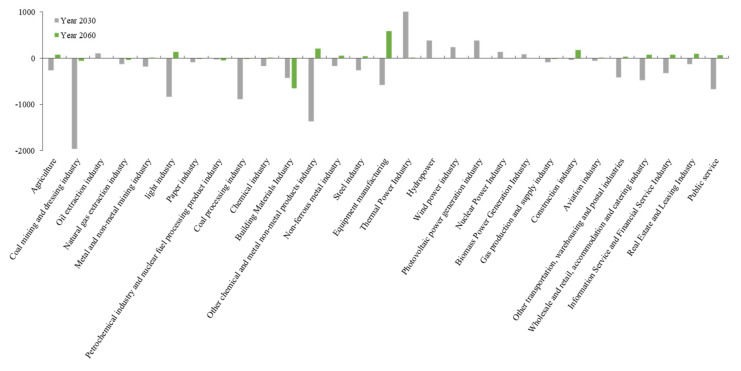
Changes in output of various industries compared with BAU under S2 scenarios in 2030 and 2060.

The industrial output significantly changed compared to the benchmark situation. Moreover, the output of non-thermal power generation, such as photovoltaic, hydro-, wind, and nuclear power will significantly expand in 2030, while coal processing and mining will significantly shrink. Further, the proportion of output among the agricultural, forestry, animal husbandry and fishery, public services, and light industries significantly decreased over time. The proportion of output for equipment manufacturing, real estate and leasing, and information and financial services increased annually (Figure 9 and Figure 10).

**Figure 11:**
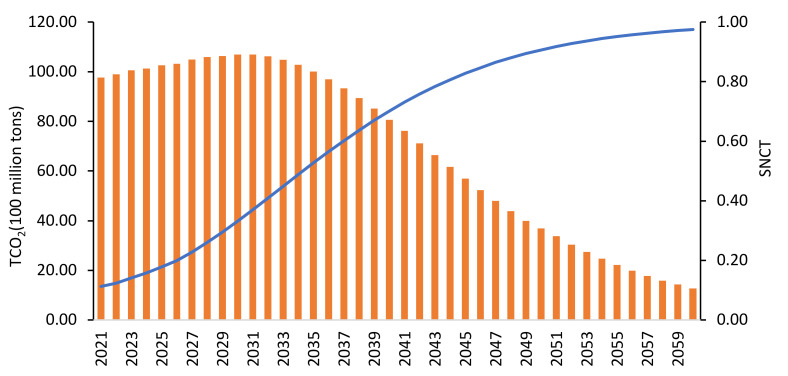
Carbon emissions and technological progress under S2 scenarios. Note: SNCT is a value between 0 and 1.

Compared with the “J”-shaped growth of carbon price under the S1 scenario, the carbon price under the S2 scenario shows an inverted “U”-shaped change with the development of climate-friendly technologies. The carbon price will rise year by year from the initial 10 CNY/ton to 140 CNY/ton in 2030, after which the carbon price will begin to decline steadily. As shown in Figure 11, when carbon prices peak, carbon emissions will also peak at 10.9 billion tons. The climate-friendly technology curve grows in an “S” shape, with a value of SNCT of 0.32 in 2030 and 0.99 in 2060. Investment, as an endogenous variable of the S-shaped technology curve, is the key to promoting technological progress. The model estimates that green technology R&D investment will account for about 2% of GDP, and it will increase year by year, helping R&D technology to cross the laboratory stage of the S-curve and use it for commercial applicato0-ions.

According to Table 3, the carbon price policy (S1 scenario) has little impact on the energy consumption structure. Fossil energy consumption will still dominate, and new energy consumption will slightly increase. However, the energy structure of the S2 scenario has significantly changed, with fossil energy gradually being withdrawn from the market, and new energy gradually monopolizing the energy consumption market.

### 5.4. Sensitivity Analysis of Technical Curve

**Figure 12:**
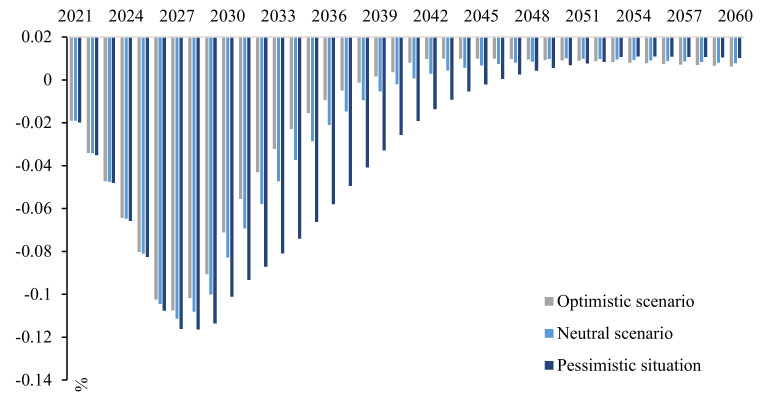
Changes in GDP of different technology curves.

It is assumed that the climate-friendly technology curve in the S2 scenario is the benchmark prospect of technological progress, and the pessimistic prospect refers to the situation in which the technological development is less than expected or difficult to commercialize. Intuitively, the technology curve in this scenario is flatter than the benchmark curve: it stays in the laboratory for a longer duration, with high resistance to the large-scale application of technology, or the technology’s permeability is low (Figure 12). In reality, it corresponds to advanced and uncertain technologies, such as hydrogen energy and carbon capture, among others, while the optimistic outlook presents the opposite characteristics.

**Figure 13:**
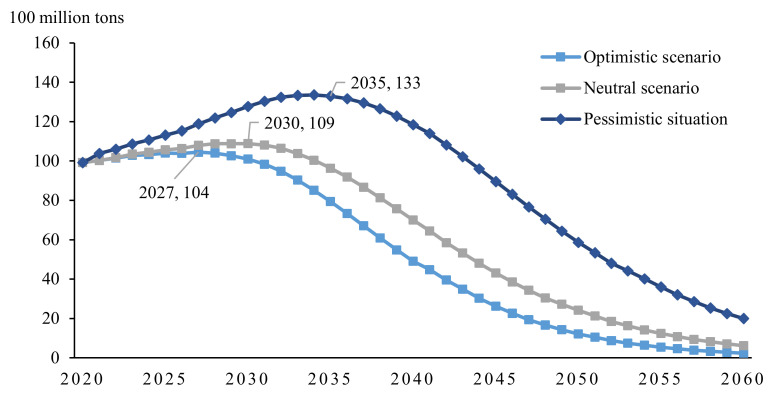
Carbon emission curve of different technologies.

As Figure 13 illustrates, the GDP under the pessimistic technology outlook experiences a relatively large negative impact, and the overall carbon emission curve increases, with a later, higher peak. Optimistic prospects provide the opposite results, but it is difficult to achieve such rapid technological development.

In short, regardless of the speed of technological development, the shape of the carbon emissions curve can change, and inflection points can appear. The only difference is the time at which peak carbon and carbon neutrality occur. Only technological progress can meet the dual constraints of carbon emissions and economic development.

## 6. Conclusions

Based on the energy-environment-economy triple-coupling (3E-CGE) model, we endogenously generate climate-friendly technologies into the model’s analysis framework by depicting the logic curve in the technology’s full life cycle and modify the energy and carbon emissions modules within the CGE model. Based on the general equilibrium analysis of this CGE model, we can draw the following conclusions and insights.

First, regarding the simulation of peak carbon and carbon neutralization results, the endogenous CGE model significantly differs from the exogenous CGE model, especially in the long-term. Compared with other results, we note that the endogenous CGE model is more reasonable in optimistic, neutral, and pessimistic technological prospects.

Second, the most appropriate development path for China involves a combination of carbon tax and carbon trading policies while steadily developing climate-friendly technologies. This includes implementing a uniform carbon tax for all industries at 100 CNY per ton, and carbon trading in eight high-emissions industries with increasing carbon prices before the peak occurs. Moreover, climate-friendly technologies, which have begun to develop steadily since 2017, should mature in 2047. On the optimal path, the country peaks in 2030 at 10.9 billion tons, then decreases annually to reach net-zero carbon emissions in 2060; additionally, the dual effects of economic growth and carbon neutrality are achieved, with a stable economic growth rate of 2.4% in the later years.

Third, climate-friendly technologies play an important role in achieving China’s goal of peak carbon in 2030 and carbon neutrality by 2060. The progress of climate-friendly technologies will gradually decrease the total carbon emissions from 2030, and will converge to a net zero in 2060, or approximately 60 million tons. The development of climate-friendly technologies will profoundly impact China’s economic structure.

The model’s results demonstrate that developing climate-friendly technologies gradually evolves the economic structure, from industry-dominated to emerging industry-dominated. Output from the new energy and service industries has rapidly expanded in the past 40 years. Meanwhile, in creating endogeneity among technologies, we can fully describe the interactions among technologies, investments, and carbon emissions. Although developing climate-friendly technologies requires substantial investments, this also stimulates economic growth and creates a mutually beneficial situation between economic growth and environmental improvements.

Regarding the traditional means of reducing emissions, such as carbon taxes and trading, carbon-pricing policies can quickly reduce the carbon emissions of energy-intensive industries in the short-term but will cause economic losses. Any economic recession will worsen as carbon pricing increases. Therefore, it is only theoretically feasible to use carbon pricing to achieve carbon neutrality. A carbon price of up to thousands of CNY per ton is an unbearable pressure for all industries and will inevitably collapse the economy. We also simulated and compared the benefits of carbon tax and carbon trading. Under the same emissions reduction target, carbon trading alone is better for the economy than only levying carbon taxes, as the former will result in less economic damage.

Collectively, carbon neutrality is not only an environmental governance issue, but also involves all aspects of society. It involves profound changes affecting all of Chinese society, including large-scale arrangements and advance planning. The government can begin with the carbon-trading market and gradually expand the scope to include all industries. Simultaneously, various climate-friendly technologies require more precise calculations in terms of their development level and potential. The government can provide preferential policies for investment in climate-friendly technologies, increase the R&D and promotion of such technologies, guide the upgrading of industrial production technologies, promote the formation of an economically beneficial zero-emissions production capacity, and intensify efforts to phase out existing high-carbon emissions assets. Moreover, a company’s improved financial performance is important in a low-carbon economy, with such positive results as increasing companies’ return on assets [57].

The endogenous technology advancement CGE model used in this article is a real economic model established based on an input-output table. It only introduces a curve for climate-friendly technology that covers all industries and does not describe the technology in detail. We can combine more knowledge with capital flow statements and data on the levels of climate-friendly technological development, capital growth rate, and depreciation rate among various industries. In doing so, we can further optimize the model and introduce a financial module to integrate both real and virtual economies. We can conduct more simulations on green credit and examine its effects on technological progress, dual-carbon goals, and economic growth in order to bring the model’s results closer to reality.

## Figures and Tables

**Table 1:** Macro SAM table.

Unit: 100 Million Chinese Yuan (Calculated Based on the Producer Price of the Year)											
	Expenditure	Production Activities	Product	Labor	Capital	Household	Government	Tax	ROW	Investment	SUM
Income											
Production Activities			2,257,733								2,257,734
Product		1,434,518				324,546	125,341		163,847	369,146	2,417,397
Labor		423,268									423,268
Capital		304,969									304,969
Household				423,268	304,969		45,615				773,852
Government								133,372			133,372
Tax		94,978	26,431			11,961					133,372
ROW			133,232							30,615	163,847
Investment						437,345	−37,584			5377	405,138
SUM		2,257,733	2,417,397	423,268	304,969	773,852	133372	133,372	163,847	405,138	7,012,948

**Table 2:** Scenario setting.

Scenario Category	Scenario Code	Setting
BAU	No exogenous intervention	The GDP growth rate drops uniformly from 6% in 2019; the compound GDP growth rate from 2020 to 2030 will be 5%, and the compound annual GDP growth rate from 2040 to 2060 will be 3%.
Carbon price policy	S1.a Carbon tax	Combined with the profitability and model tests of various domestic industries, we set a unified tax rate for the whole society and the tax rate increases year by year. The maximum carbon tax rate will not exceed 1800 CNY/ton. ^1^
	S1.b Carbon trading	According to the “the current Guangdong Province’s Implementation Plan for the Allocation of Carbon Emission Allowances in 2020”, the model sets the industries for carbon trading as: petrochemicals, chemicals, building materials, steel, non-ferrous metals, papermaking, electricity, aviation and their respective free carbon emission allowances.
	S1 Carbon Tax + Carbon Trading	Carbon tax and carbon trading implemented simultaneously
Technological progress	S2.a	Optimistic prospect of technology development
	S2.b	Pessimistic prospect of technology development
	S2	S1+ neutral prospect of technology development Technology curve

^1^: Countries that have levied carbon taxes currently have a carbon tax rate of approximately CNY 80–800 per ton of carbon, but most of them are developed countries.

**Table 3:** The proportion of energy consumption under the S1 scenario & S2 scenario.

	S1			S2		
	2020	2030	2060	2020	2030	2060
Coal	0.1%	10%	11.2%	49.3%	40.6%	2.9%
Oil	49.6%	47.7%	45%	8.4%	8.3%	0.8%
Natural Gas	4.4%	4.3%	4.3%	3.1%	2.9%	0.3%
Thermal Power	0.8%	0.9%	1%	23.3%	20.9%	1.5%
New Energy Power	35.1%	37.1%	38.5%	16%	27.4%	94.5%

## Data Availability

The data used to support the findings of this study are available from the corresponding author upon request.
